# Supplementary material for: Predation and fragmentation portrayed in the statistical structure of prey time series
Source: BMC Ecol. 2009 May 6;9:10. doi: 10.1186/1472-6785-9-10 (PMC2689204; doi:10.1186/1472-6785-9-10)
Supplement: Additional file 2 — Voles and related classes ODDox Documentation. ODDox documentation of the agent-based model (ALMaSS) applied by Hendrichsen et al. The documentation is started by activating main.html. [file 1472-6785-9-10-S2.zip › Vole_ODDox/functions_vars_0x70.html]

ALMaSS ODDox: Class Members - Variables

- Main Page
- Related Pages
- Classes
- Files

- Alphabetical List
- Class List
- Class Hierarchy
- Class Members

- All
- Functions
- Variables

- a
- b
- c
- f
- g
- h
- i
- j
- l
- m
- n
- p
- r
- s
- t
- v
- x
- y

### - p -

- Percent
  : Rotation
  , Starter- PM
    : struct\_Predator- PreyResponse1
      : TPredator- PreyResponse2
        : TPredator- ProbesSet
          : Population\_Manager

---

Generated on Thu Jan 22 14:13:45 2009 for ALMaSS ODDox by 
 1.5.6
